# Liver function recovery of COVID-19 patients after discharge, a follow-up study

**DOI:** 10.7150/ijms.50691

**Published:** 2021-01-01

**Authors:** Ya-Wen An, Shuo Song, Wei-Xin Li, Yong-Xin Chen, Xiao-Peng Hu, Jia Zhao, Zhi-Wen Li, Guang-Yu Jiang, Cheng Wang, Jian-Chun Wang, Bo Yuan, Han-Qing Liu

**Affiliations:** 1 Central laboratory, Shenzhen Samii Medical Center, Shenzhen city, Guangdong province, China, 518118.; 2 Neurology department, Shenzhen Samii Medical Center, Shenzhen city, Guangdong province, China, 518118.

**Keywords:** COVID-19 pneumonia, liver injury, discharge management, follow-up

## Abstract

**Objective:** The aim of this study was to observe the liver function recovery of COVID-19 patients after discharge.

**Patients and Methods:** A total of 253 discharged COVID-19 patients in Shenzhen city, China were selected. The clinical characteristics of these patients were assessed. A 2-month follow-up and laboratory hematology test were performed to examine the status of patients' liver function.

**Results:** Patients combined with liver diseases, especially fatty liver, are more likely to progress to severe condition (*P<*0.05). Patients in severe condition and those with liver diseases have higher rates of liver injuries during hospitalization, characterized by a significant increase in alanine aminotransferase (ALT) and aspartate aminotransferase (AST, *P<*0.01). The ALT, AST/ALT, gamma-glutamyl transferase (GGT), alkaline phosphatase (ALP), total protein (TP), albumin (ALB), and A/G levels showed significant differences in comparison with the control group (*P<*0.05, and *P<*0.001); and the outlier ratio of A/G, ALT, GGT and ALP of patients remained abnormal higher within 14 days after discharge (*P<*0.001). Liver injuries of COVID-19 patients may be related to the epidemiological characteristics, clinical indexes, basic diseases, symptoms, drug treatment during hospitalization and the complications. Indicators of liver function were correlated with cardiac function, renal function, thyroid function, lipid metabolism, glucose metabolism, immune index, leukocyte, erythrocyte, hemoglobin and platelet related indexes. The outlier ratio of TP, ALB and GLB remained extremely low throughout the follow-up period; the outlier ratio of ALT, AST and GGT decreased below 10% from a high level at 40 days after discharged. However, the outlier ratio of A/G, AST/ALT and ALP remained high during the follow-up period.

**Conclusions**: Abnormal liver function might indicate worse recovery of COVID-19 patients. Changes in liver function should be emphasized during long-term follow-up of COVID-19 patients after hospital discharge; the necessity of employing appropriate interventions for liver function repair should be emphasized.

## Introduction

The outbreak of SARS-Cov-2 has been a significant public health threat over the past few months 1. Worldwide, the number of laboratory-confirmed patients infected with COVID-19 at the end of October 12 2020 was over 37 million and more than 1 million patients died. The overall case fatality rate exceeded 5%, and the rates are higher in older patients, men, and those with basic disease 2-4. The virus has been shown to affect multiple organs and systems, such as the heart, kidneys, liver, nervous system and blood system 5, 6, however, the impacts on the reproductive system and whether there is vertical transmission between mother and child is still controversial 7. Patients with diabetes, hypertension, cancers, cardiopathy, nephropathy and liver diseases under a higher risk of becoming severely conditions or even dying once infected. Besides, they have to weigh risk and benefit of giving treatment *vs* chances of getting infected, especially for cancer patients 8.

Recently, COVID-19 patients with liver diseases during hospitalization have received particular attention, based on the experience from the previous SARS epidemic 9. Previous studies indicated that 2%-11% patients with COVID-19 had underlying chronic liver diseases 10. The clinical implications of liver derangement such as chronic hepatitis B or hepatitis C, non-alcoholic fatty liver disease, liver cirrhosis, and liver transplantation might be variable in different clinical scenarios, and proper management should be provided to COVID-19 patients with liver diseases 11.

About 60% of patients infected with SARS have been reported with liver impairments. In the current COVID-19 pandemic, the prevalence of liver injury and associated clinical characteristics are not well-defined. Deaths due to liver damage have been reported in COVID-19 patients 12, and the effects of COVID-19 in those with liver diseases and liver injuries are worse, resulting in the impending global economic crisis 13. Hepatic function injuries are mainly characterized by acute increase in the ALT and AST levels during hospitalization^14^; however, the possible mechanisms are still unclear and might be attributed to the immune mediated damage, direct cytotoxicity, anoxia, drug-induced liver injury and reactivation of pre-existing liver disease^3^, ^10^, ^15^.

A surveillance of viral clearance in the liver and the long-term outcome of COVID-19 is required. Hundreds of studies have been conducted to examine COVID-19 patients who developed liver diseases and hepatic impairment during hospitalization; however, the duration and returning cycle of patients with liver damages after discharge are still unclear. More than 28 million COVID-19 patients have been discharged from hospital so far. The follow-up study, especially observation of recovery of complications and sequelae, is very important to help us understand the pathogenesis and treatment process of COVID-19. In the present study, 253 discharged COVID-19 patients in Shenzhen City, China, were followed-up for more than 2 months, and some of them underwent one or more hematological tests including liver function test. The causes and functional recovery time of the abnormal liver were revealed, and these might provide clinically helpful references for the management of COVID-19 patients.

## Methods

### Patients and controls

This was a cross-sectional study among discharged COVID-19 patients recruited from the Shenzhen Samii Medical Center (also named The Fourth People's Hospital of Shenzhen), which is the only referral quarantine place for discharged COVID-19 patients in Shenzhen, China, since February 22, 2020. There were 417 patients diagnosed with COVID-19 based on the World Health Organization interim guidance from January 11, 2020 to February 21 and treated in the Third People's Hospital of Shenzhen 16. Only 3 out of the 417 patients died of COVID-19 during hospitalization. A total of 253 of the 417 patients were sent to Shenzhen Samii Medical Center for a 14-day medical observation and follow up after discharge from the Third People's Hospital of Shenzhen 17. During the observation period, all patients underwent voluntary and free medical examination in order to determine their health status. The physical examination items included blood routine, blood biochemistry, urine routine, stool routine and chest CT scans. The patient can choose one or several items freely. Of the 253 patients, 163 underwent hematologic detection within 14 days after their first discharged, and the other 90 patients didn't take hematologic detection within 14 days. A total of 46 of the 163 patients received at least 4 more detection from 14 days to 2 months after they discharge. We collected all the test results to analyze the changes in their serum hepatic function indicators. Data on the serum hepatic function indicators of randomly selected 152 healthy persons who were never infected with SARS-Cov-2 and underwent the same detection test to assess their liver function during the follow-up (from February 22 to May 27, 2020) were collected as the control group. The division of severe and non-severe group of COVID-19 patients was according to the Diagnosis and treatment of pneumonia caused by novel coronavirus (trial version 7).

### Examining and analyzing the indicators

The clinical characteristics, treatment during hospitalization and compilations of the discharge summary of the 253 patients described above were collected to examine the influences of basic liver disorders on the progression of disease and the impacts of COVID-19 in patient's liver function. The liver disease referred to the COVID-19 patients who developed basic diseases including hepatitis B, fatty liver, hepatic cyst, and cholecystopathy prior to acquiring the infection. The liver injury referred to the COVID-19 patients was defined by elevation of liver enzymes. A total of 3 mL of blood was extracted from every patient while on empty stomach, and the serum was separated by centrifugation at 3,000 r/min for 15 minutes. The serum biochemistry detection was performed using the automatic biochemical analyzer (C501, Roche, Germany) and matching reagents.

### Ethics statement

This study was approved by the Ethics Committee of Shenzhen Samii Medical Center (approval number: SSMC-R-20200401), and a written informed consent was signed by all patients and healthy volunteers.

### Statistical analysis

All analyses were conducted using the SPSS software version 19.0. Continuous variables were expressed as means ± SD, and the independent sample T-test (T_2_, assumed homogeneity of variance) was used to compare the intergroup differences. Categorical variables were compared using the Chi-square test (Monte Carlo Sig. T_2_). Binary logistics regression was used to analyze the independent impact factors, while the bivariate Pearson test was used to analyze the correlation between the two indexes. A *P* value of <0.05 was considered significant.

## Results

The epidemiological characteristics of the 253 discharged COVID-19 patients are shown in **Table 1.** Of them, 140 were women and 113 were men; the patients' age was 45.3 ± 17.8 (range: 1-86, median: 47) years; of the total patients, 58 (22.9%) had a severe condition during hospitalization, while 92 patients experienced 1 or more recurrence (22.2%, 92/414). There were significant differences in age, gender, underlying diseases and symptoms between severe and non-severe patients during hospitalization (*P<*0.05, *P<*0.01, and *P<*0.001, **Table 1**). Age and time from onset to admission were independent risk factors for severe patients (*P<*0.05, **Table 1**). The results of binary Logistic regression showed that the *P* value of blocked or watery nose was less than 0.01, however, there were only 10 patients in total with the symptom of blocked or watery nose, which may lead to statistical bias. Therefore, whether the index of blocked or watery nose can be identified as an independent risk factor for severe patients deserves further discussion.

Of the 253 patients, 51 (20.2%) had liver diseases prior to acquiring COVID-19, including hepatitis B (15, 5.9%), fatty liver (22, 8.7%), hepatic cyst (14, 5.5%), and cholecystopathy (5, 2.0%); patients in severe condition during hospitalization had significantly higher probabilities to carry liver diseases, especially fatty liver, than patients in non-severe condition (29.3 *vs* 17.4, *P<*0.05; 15.5% *vs* 6.7%, *P<*0.05, **Table 1**, Figure S1). Patients with liver diseases were significantly older; had significantly longer hospital stays, and higher incidence of severe condition (*P<*0.05, and *P<*0.001, **Table 1**).

The clinical characteristics of the 253 COVID-19 patients during hospitalization are presented in Table 2. There were significant differences in most of clinical indicators between severe and non-severe patients (*P<*0.05, *P<*0.01, and *P<*0.001, **Table 2**). The oxygenation index of severe patients was much lower than that of non-severe patients (424.4 mmHg *vs* 464.8 mmHg), however, there was no statistically significant difference due to the large standard deviation. Breathe, NEUT (%), LYM (%), PCT and D-dimer were independent risk factors for severe patients (*P<*0.05, *P<*0.01, and *P<*0.001, **Table 2**). The ALT and AST levels of patients who were in severe condition or had basic liver diseases were significantly higher during hospitalization than other patients (*P<*0.01, and *P<*0.001, **Table 2**). The WBC and LYM# levels of patients with liver diseases were significantly lower than those without liver diseases (*P<*0.05, and *P<*0.01, **Table 2**).

The treatments provided to all 253 COVID-19 patients during hospitalization and their complications noted in the discharge summary are presented into Tables 3 and 4, respectively. The application of oxygen inhalation, antiviral drugs, anti-infection drugs, vasoactive drugs, hormonotherapy, immunoregulatory drugs, drugs to regulate intestinal flora, and symptomatic treatment drugs were markedly higher in patients under severe condition (*P<*0.05, *P<*0.01, and *P<*0.001, **Table 3**). The application of oxygen inhalation and arbidol were over 10% higher in patients with liver diseases than in those without liver diseases. The application of interferon was nearly 10% lower in patients with liver diseases. Moreover, application of drugs to regulate intestinal flora, acetylcysteine and stomach-protective drug was much higher in patients with liver diseases than in those without liver diseases (*P<*0.05, **Table 3**). None of the patients received tocilizumab or remdesivir. The differences in drug use may be due to the different proportion of patients with severe condition; this finding also indicated that treatment is one of the primary causes of liver damage 18-20.

Patients under severe condition have a higher risk of developing complications (*P<*0.05, *P<*0.01, and *P<*0.001, **Table 4**). The incidence of complications including hematological changes, hypokalemia, thrombus, cardiovascular injury, respiratory injury, and lung injury, were significantly higher in patients with liver diseases than in those without liver diseases (*P<*0.05, and *P<*0.01, **Table 4**).

The serological hepatic function index of 163 of 253 COVID-19 patients within 14 days after discharge were compared with that of healthy persons who were never infected with SARS-Cov-2. No significantly differences were observed in the baseline characteristics between discharged patients and healthy persons by gender (51.5% *vs* 52.0%). Moreover, the discharged patients were slightly older than the control group (50.5 *vs* 47.7 years). The duration time of hepatic function detection was 7.5 ± 3.5 (range: 1-14) days after discharge. The TP, ALB, A/G and AST/ALT levels of discharged patients were significantly lower than those of healthy persons (*P<*0.05, *P<*0.01, and *P<*0.001); and the ALT, GGT and ALP levels of discharged patients were significantly elevated (*P<*0.001, **Table 5**). The outlier ratio of A/G, ALT, GGT and ALP were significantly higher in discharged patients than in the control group (*P<*0.01, and *P<*0.001, **Table 5**). The TP and ALB levels of patients in severe condition were significantly lower than that of patients in non-severe condition (*P<*0.01, **Table 5**); and the GGT levels and outlier ratio of patients in severe condition were significantly elevated (*P<*0.01, and *P<*0.001, **Table 5**).

**Figure 1:**
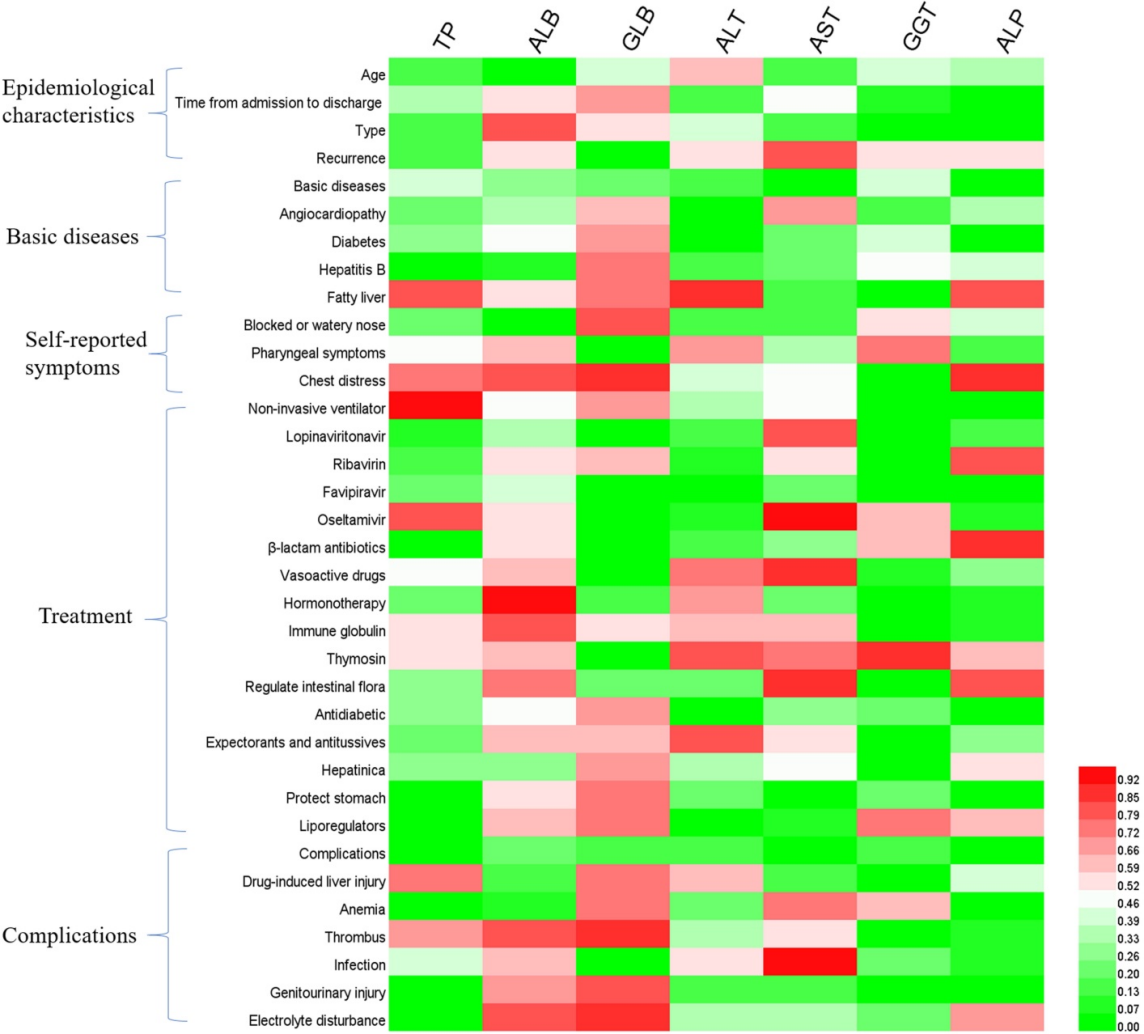
Binary logistics regression analysis of hepatic function indexes and clinical characteristics of patients. *P<*0.05 was considered as the independent impact factor.

**Figure 2:**
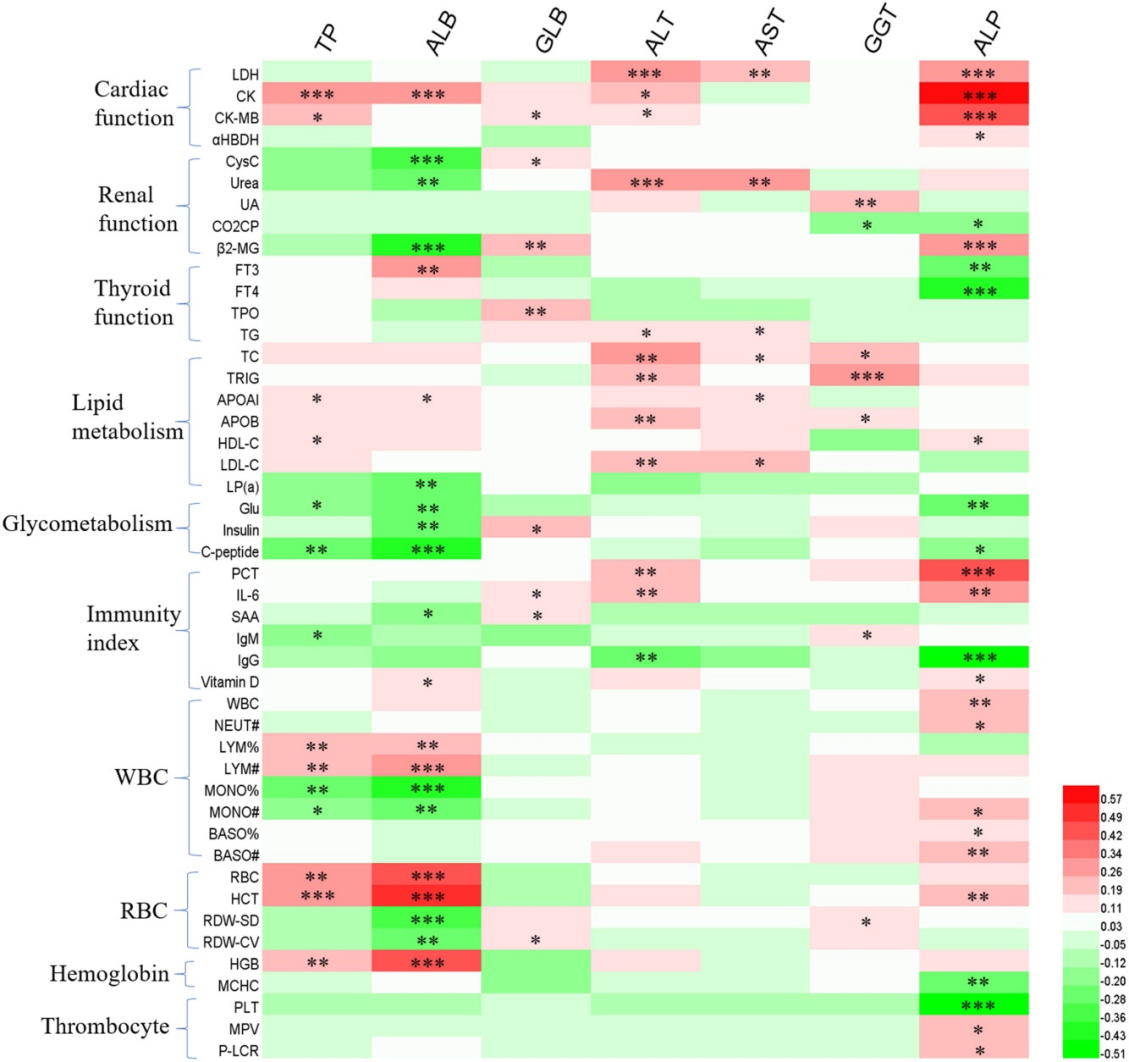
The correlation between hepatic enzymes and other hematologic indicators. Red and green represents positive and negative correlation, respectively. The significance *P<0.05, **P<0.01, ***P<0.001.

In order to further explore the causes of liver function disorder during the follow-up period, we analyzed the effects of patients' clinical characteristics on liver function indexes (**Figure 1**), as well as the correlation between liver function indexes and other hematologic indicators (**Figure 2**). The results of binary logistics regression analysis of clinical data showed that the independent influencing factors of at least one liver function index covered almost all the clinical characteristics, treatment during hospitalization and complications (**Figure 1**). The results of correlation analysis showed that the levels of liver function indexes were positively or negatively correlated in a very complex way with cardiac function indexes, renal function indexes, thyroid function indexes, lipid metabolism indexes, glucometabolic indexes, immunity indexes and blood routine indexes (**Figure 2**).

The liver function of 46 COVID-19 patients who underwent at least four tests 14 days to 2 months after discharge were observed; the details of the 46 patients are described in Figure S2. The age of the 46 patients was 46.8 ± 15.3 (range: 17-86 and median: 48) years. Of them, 20 (43.5%) were women and 10 (21.7%) had severe condition during hospitalization. The average levels of the TP, ALB and A/G in COVID-19 patients, which was significantly lower than the control group within 14 days after discharge, elevated gradually; the average levels of the ALT, GGT and ALP, which was significantly higher in patients than the control group within 14 days after discharge, decreased gradually (*P<*0.05, and *P<*0.01, **Table 6**). The outlier ratio of TP, ALB, and GLB remained extremely low. The outlier ratio of ALT, AST and GGT continued to decline and was less than 10% during the 4^th^ test (*P<*0.05, and *P<*0.01, **Table 6**). The outlier ratio of A/G, AST/ALT and ALP remained high on all the four tests (**Table 6**).

## Discussion

More than 28 million of COVID-19 patients have overcome the disease, and are gradually returning to work and social life. Nonetheless, COVID-19 may cause further downstream issues in these patients, such as possible reactivation of the virus, complications and sequelae, and post-traumatic stress disorder 21. One of the main purposes of follow-up is to monitor nucleic acid reactivation. Although the current evidences suggest that positivity on follow-up RT-PCR not imply infectivity 22, typical symptoms occur in a significant proportion of recurrence patients and require further treatment 23-25. On the other hand, it is imperative to understand the possible outcome of discharged COVID-19 patients, especially if they have any other detrimental illnesses by longitudinal analysis to safeguard their life in future 26, 27.

In the present study, we reviewed the clinical characteristic of 253 out of 417 COVID-19 patients confirmed from January 11 to February 21 in Shenzhen City, China. The abnormal liver function of these 417 patients during hospitalization was reported earlier 16. Of them, 76.3% had abnormal liver test results and 21.5% had liver injury during hospitalization; moreover, 23.4%, 14.8%, 11.5% and 24.4% of these patients had extremely elevated ALT, AST, total bilirubin, and GGT levels. Unlike the previous studies, the present study focused on the 253 discharged patients, especially those who carried chronic liver diseases before infected by the SRAS-Cov-2, and those who with liver injuries, so as to better explore the causes of liver injuries and its recovery progress. The results showed that the 20.2% of the patients had liver diseases before acquiring COVID-19. The major liver diseases included hepatitis B, fatty liver, hepatic cyst and cholecystopathy. Patients with severe condition during hospitalization had significantly higher probabilities of carrying liver diseases, and had significantly higher probabilities to be diagnosed with liver injuries than those with non-severe cases. The proportion of patients with liver diseases in the present study is higher than in previous studies, perhaps because we adjusted patients diagnosed with chronic liver disease such as hepatitis B, fatty liver, hepatic cyst, *etc.* after admission to those with underlying liver diseases.

Many recent studies suggested that COVID-19 patients with concurrent liver diseases may have a higher rate of progression to severe condition and even a higher mortality rate 28, 29. In a multicenter retrospective study by Iavarone *et al.* 30, patients with cirrhosis and COVID-19 were found to have high rates of 30-day mortality. However, many other people hold more optimistic views. Liu 31 considered that the association between liver injury indicators and mortality should be interpreted cautiously. Bangash *et al.* 32 considered that clinically significant liver injury is uncommon, even in severely ill patients, and there is little cause for concern. Our results showed that patients with liver diseases were significantly older than the other patients, and had more basic diseases. The ALT and AST levels of patients in severe condition or those with basic liver diseases were much higher than those of other patients. This finding is consistent with those of other previous studies 33. The PCT and D-dimer levels of patients in severe condition were significantly higher than those of patients in non-severe condition; however, their NEUT and LYM levels were significantly lower. The WBC and LYM levels of patients with liver diseases were significantly lower than those without liver diseases. Whereas PCT and D-dimer are considered as risk factors for mortality in patients with COVID-19; patients with D-dimer levels ≥2.0 µg/mL had a higher incidence of mortality compared with those with D-dimer levels <2.0 µg/mL 34. The WBC decrease, especially of LYM and NEUT, is one of the most common laboratory abnormalities in patients with COVID-19.

In addition, patients in severe condition had significantly higher incidence of developing complications than those in non-severe condition, including drug-induced liver injury, hematologic complications, cardiovascular injury, respiratory injury, infection, lung injury, genitourinary injury, steroid-induced diabetes, and electrolyte disturbance. Liver disease was associated with hematologic complications, hypokalemia, thrombus, cardiovascular injury, respiratory injury and lung injury. These complications were proved to be associated with fatal outcome of COVID-19 27, 35, 36. The present results suggest the risk of liver disease in COVID-19 patients. Whether an association exist between liver enzyme abnormalities and mortality or not, it is still important to strengthen the detection and monitoring during the clinical and follow-up period.

Overall, there was no significant difference in physicotherapeutics and medication-based treatment between patients with liver disease and those without liver disease. However, the application of oxygen inhalation, antiviral drugs, anti-infection drugs, vasoactive drugs, hormonotherapy, immunoregulatory drugs, drugs to regulate intestinal flora, and symptomatic treatment drugs were markedly higher in patients under severe condition. Many of these drugs have been shown to be hepatotoxic, and the side effects of drugs may be one of the primary causes of liver damage in COVID-19 patients, especially for those in severe condition 19, 20, 37.

The serological hepatic function indexes of 163 out of the 253 COVID-19 patients within 14 days after discharge were compared with that of healthy persons who were never infected with SARS-Cov-2. The levels of TP, ALB, A/G and AST/ALT in patients were significantly lower than those of the control group, and their ALT, GGT and ALP levels were significantly higher. These results indicated that the liver function still not been restored within approximately 1 week (7.5±3.5 days) from the time of discharge. The outlier ratio of A/G, ALT, GGT and ALP was significantly higher in these patients than in the control group. For patients with liver diseases, the levels and outlier ratio of GGT were higher than those in patients without liver diseases. These results suggest that a 14-day follow-up is not sufficient to determine liver recovery time and longer follow-up period is necessary. To further observe the recovery of liver function, 46 patients with COVID-19 underwent at least four tests from 14 days to 2 months after discharge were selected. The outlier ratio of TP, ALB, and GLB remained extremely low. The outlier ratio of ALT, AST, and GGT continued to decline and was less than 10% at the 4^th^ test.

In conclusion, chronic liver disease, especially fatty liver, may increase the risk of severe COVID-19 disease. Liver injury during hospitalization was characterized by abnormalities in ALT and AST levels, and may be associated with epidemiological characteristics, clinical indicators, therapeutic agents, and other complications. Liver injury within 14 days after discharged was primarily manifested by an abnormal increase of ALT, GGT and ALP levels. The outlier ratio of ALT, AST, and GGT recovered to below 10% at 40 days after discharged. The factors affecting liver function were very complex. It might be correlated with epidemiological characteristics, clinical characteristics, therapeutic drugs, complications and other test indicators, such as cardiac function indicators, renal function, thyroid function indicators, immunity, leukocytes, erythrocytes and coagulation indicators. Changes in liver function should be emphasized during a long-term follow-up (at least 2 months) for COVID-19 patients after discharge and appropriate interventions and liver function repair are necessary.

The present study can help peoples to understand the progression of COVID-19, particularly the impacts on liver function, and provide references to the treatment of patients during hospitalization and management after discharge. However, this study also has some limits. First of all, some patients were unwilling to undergo laboratory tests when no nucleic acid positive results were reported in the follow-up examination, therefore, only 163 of the 253 patients underwent hematologic examinations in 14 days after discharged, and the patients number had dropped further to 46 within two months who had more than four tests; hence, further follow-up observation using a large sample size is necessary. Besides, some other indicators of liver function, such as bilirubin, have been proved to show significant differences between severe and non-severe patients 38, but these indicators were not tested for the patients included in this study. In subsequent follow-up, additional tests of relevant liver function indicators like bilirubin should be added to provide more evidences.

## Supplementary Material

Supplementary figures.

## Figures and Tables

**Table 1:** Epidemiological characteristics of 253 COVID-19 patients

Epidemiological characteristics	Total (N=253)	Non-severe (N=195)	Severe (N=58)	Logistic	Without liver disease (N=202)	With liver disease (N=51)
Gender: Female (No., %)	140 (55.3)	117 (60.0)	23 (44.8) **	0.378	112(55.4)	28(54.9)
Age (years)	45.3±17.8	41.9±17.8	56.8±12.1***	0.013	43.5±18.1	52.5±14.5^^^
Time from onset to admission (days)	4.2±3.9	3.9±3.9	5.4±3.9*	0.044	4.3±3.9	3.9±4.1
Time from admission to discharge (days)	26.2±10.0	23.8±8.7	34.1±10.4***	0.061	25.5±10.2	29.1±9.0^
Type: non-severe (No., %)	195 (77.1)	/	/	/	161(79.7)	34(66.7) ^
Recurrence (No., %)	92 (36.4)	79 (40.5)	13 (22.4) *	/	76(37.6)	16(31.4)
Family cluster (No., %)	155 (61.3)	117 (60.0)	38 (65.5)	0.640	122(60.4)	33(64.7)
Exposure to Wuhan (No., %)	106 (41.9)	75 (38.5)	31 (53.4) *	0.104	84(41.6)	22(43.1)
**Basic diseases (No., %)**						
Angiocardiopathy	37 (14.6)	18 (9.2)	19 (32.8) ***	0.416	28(13.9)	9(17.6)
Hypertension	38 (15.0)	22 (11.3)	16 (27.6) **	0.483	28(13.9)	10(19.6)
Diabetes	24 (9.5)	12 (6.2)	12 (20.7) **	0.512	19(9.4)	5(9.8)
Liver Disease	51 (20.2)	34 (17.4)	17 (29.3) *	0.413	/	/
Hepatitis B	15 (5.9)	11 (5.6)	4 (6.9)	/	/	/
Fatty liver	22 (8.7)	13 (6.7)	9 (15.5) *	/	/	/
Hepatic cyst	14 (5.5)	9 (4.6)	5 (8.6)	/	/	/
Cholecystopathy	5 (2.0)	3 (1.5)	2 (3.4)	/	/	/
Other diseases	41 (16.2)	31 (15.9)	10 (17.2)	0.979	31(15.3)	10(17.6)
Without diseases	130 (51.4)	112 (57.4)	18 (31.0) ***	0.574	130(64.4)	0(0.0) ^^^
**Self-reported symptom (No., %)**						
Fever	155 (61.3)	110 (56.4)	45 (77.6) **	0.129	124(61.4)	31(60.8)
Cough	91 (36.0)	63 (32.3)	28 (48.3) *	0.071	70(34.6)	21(41.2)
Weak	8 (3.2)	6 (3.1)	2 (3.4)	0.512	5(2.5)	3(5.9)
Blocked or watery nose	10 (4.0)	8 (4.1)	2 (3.4)	0.004	7(3.5)	3(5.9)
Pharyngeal symptoms	17 (6.7)	13 (6.7)	4 (6.9)	0.636	13(6.4)	4(7.8)
Muscle or joint pain	7 (2.8)	5 (2.6)	2 (3.4)	0.747	5(2.5)	2(3.9)
Chest distress	10 (4.0)	6 (3.1)	4 (6.9)	0.647	9(4.4)	1(2.0)
Dizziness or headache	7 (2.8)	6 (3.1)	1 (1.7)	0.572	7(3.5)	0(0.0)
Gastrointestinal symptom	4 (1.6)	3 (1.5)	1 (1.7)	0.747	4(2.0)	0(0.0)
Other symptom	6 (2.4)	3 (1.5)	3 (5.2)	0.647	4(2.0)	2(3.9)
Asymptomatic patients	41 (16.2)	40 (20.5)	1 (1.7) **	0.210	34(16.8)	7(13.7)

*P<0.05, **P<0.01, ***P<0.001, compared with the non-severe patients; ^ *P<*0.05, ^^^ *P<*0.001, compared with the patients without liver diseases.

**Table 2:** Clinical indexes of 253 COVID-19 patients during hospitalization

Characteristics	Total	Non-severe	Severe	Logistic	Without liver disease	With liver disease
Body temperature (°C)	37.1±0.7	37.0±0.6	37.6±0.8***	0.062	37.1±0.7	37.1±0.7
Breathe (N)	20.4±6.9	19.8±1.3	23.7±17.8*	0.002	19.9±1.3	22.7±16.4
Heart rate (N)	87.2±13.4	86.5±12.9	91.1±15.8	0.607	87.2±12.9	87.0±15.7
High pressure (mmHg)	125.6±14.8	123.9±14.1	135.5±15.0**	0.148	125.5±14.9	126.0±14.7
Low pressure (mmHg)	80.2±10.3	79.8±10.3	82.2±9.9	0.709	79.9±10.3	81.5±10.4
Oxygenation index (mmHg)	458.4±113.7	464.8±107.3	424.4±140.8	0.591	459.8±119.4	451.7±85.6
Oxyhemoglobin saturation (%)	98.0±1.3	98.0±1.2	97.7±2.0	0.654	98.0±1.3	97.8±1.4
ALT (U/L), Ref: women 7-40; men 9-50	34.6±49.4	32.0±48.8	44.2±51.0↑	0.674	28.9±29.5	57.5±90.9^^↑
AST (U/L), Ref: 15-40	31.1±25.0	27.8±20.9	43.6±34.3**↑	0.133	27.6±14.8	45.6±45.7^^^↑
AST/ALT, Ref: 0.5-1.5	1.3±0.8	1.3±0.8	1.3±0.8	0.316	1.3±0.8	1.2±0.8
WBC (10^9^/L), Ref: 3.5-09.5	5.4±1.9	5.4±1.9	5.1±1.9	0.897	5.5±1.9	4.9±1.8^
NEUT (%), Ref: 40-75	58.9±12.2	57.1±11.5	65.2±12.7***	0.020	58.7±12.0	59.6±13.2
NEUT# (10^9^/L), Ref: 1.8-6.3	3.3±1.6	3.2±1.6	3.5±1.9	0.633	3.4±1.7	2.9±1.5
LYM (%), Ref: 20-50	29.4±10.8	31.2±10.3	23.1±10.3***	0.019	29.8±10.7	28.0±11.6
LYM# (10^9^/L), Ref: 1.1-03.2	1.5±0.7	1.6±0.7	1.1±0.5***	0.069	1.6±0.7	1.3±0.5^^
PCT (ng/mL), Ref: 0-0.5	0.1±0.1	0.0±0.1	0.1±0.1**	0.010	0.0±0.7	0.1±0.1
D-dimer (mg/L), Ref: 0-0.5	0.5±0.8	0.4±0.3	1.1±1.6***↑	0.000	0.5±0.8	0.6±0.8

*P<0.05, **P<0.01, ***P<0.001, compared with the non-severe patients; ^ *P<*0.05, ^^ *P<*0.01, ^^^ *P<*0.001, compared with the patients without liver diseases. The symbol ↑ respected the value was significantly higher or than the normal reference range.

**Table 3:** Treatment of 253 COVID-19 patients during hospitalization

Treatment (No., %)	Total (N=253)	Non-severe (N=195)	Severe (N=58)	Without liver disease (N=202)	With liver disease (N=51)
Tracheal intubation	7 (2.8)	0 (0)	7 (12.1) ***	6 (3.0)	1 (2.0)
Non-invasive ventilator	23 (9.1)	0 (0)	23 (39.7) ***	17 (8.4)	6 (11.8)
Oxygen inhalation	119 (47.0)	68 (34.9)	51 (87.9) ***	89 (44.1)	30 (58.8)
Interferon	198 (78.3)	146 (74.9)	52 (89.7) *	162 (80.2)	36 (70.6)
Lopinaviritonavir	193 (76.3)	145 (74.4)	48 (82.8)	152 (75.2)	41 (80.4)
Arbidol	79 (31.2)	62 (31.8)	17 (29.3)	59 (29.2)	20 (39.2)
Ribavirin	49 (19.4)	30 (15.4)	19 (32.8) **	36 (17.8)	13 (25.5)
Favipiravir	38 (15.0)	29 (14.9)	9 (15.5)	29 (14.4)	9 (17.6)
Oseltamivir	22 (8.7)	10 (5.1)	12 (20.7) ***	19 (9.4)	3 (5.9)
Chloroquine	25 (9.9)	25 (12.8)	0 (0) **	21 (10.4)	4 (7.8)
Anti-infection drugs	63 (24.9)	30 (15.4)	33 (56.9) ***	50 (24.8)	13 (25.5)
Quinolones antibiotics	47 (18.6)	24 (12.3)	23 (39.7) ***	37 (18.3)	10 (19.6)
β-lactam antibiotics	24 (9.5)	6 (3.1)	18 (31.0) ***	19 (9.4)	5 (9.8)
Macrolides antibiotics	10 (4.0)	5 (2.6)	5 (8.6) *	8 (4.0)	2 (3.9)
Antifungal drugs	8 (3.2)	0 (0)	8 (13.8) ***	6 (3.0)	2 (3.9)
Vasoactive drugs	12 (4.7)	1 (0.5)	11 (19.0) ***	10 (5.0)	2 (3.9)
Hormonotherapy	64 (25.3)	20 (10.3)	44 (75.9) ***	49 (24.3)	15 (29.4)
Immunoregulation	107 (42.3)	58 (29.7)	49 (84.5) ***	82 (40.6)	25 (49.0)
Thymalfasin	76 (30.0)	44 (22.6)	32 (55.2) ***	60 (29.7)	16 (31.4)
Immune globulin	56 (22.1)	17 (8.7)	39 (67.2) ***	43 (21.3)	13 (25.5)
Thymosin	16 (6.3)	8 (4.1)	8 (13.8) **	11 (5.4)	5 (9.8)
Traditional Chinese medicine	78 (30.8)	64 (32.8)	14 (24.1)	60 (29.7)	18 (35.3)
Regulate intestinal flora	93 (36.8)	63 (32.3)	30 (51.7) **	68 (33.7)	25 (49.0) ^
Acetylcysteine	80 (31.6)	48 (24.6)	32 (55.2) ***	58 (28.7)	22 (43.1) ^
Antidiabetic	25 (9.9)	12 (6.2)	13 (22.4) ***	19 (9.4)	6 (11.8)
Hypotensor	42 (16.6)	25 (12.8)	17 (29.3) **	32 (15.8)	10 (19.6)
Expectorants and antitussives	41 (16.2)	20 (10.3)	21 (36.2) ***	31 (15.3)	10 (19.6)
Hepatinica	23 (9.1)	17 (8.7)	6 (10.3)	17 (8.4)	6 (11.8)
Protect stomach	22 (8.7)	10 (5.1)	12 (20.7) ***	13 (6.4)	9 (17.6) ^
Liporegulators	12 (4.7)	4 (2.1)	8 (13.8) ***	7 (3.5)	5 (9.8)
Psychotropic drug	10 (4.0)	7 (3.6)	3 (5.2)	6 (3.0)	4 (7.8)

*P<0.05, **P<0.01, ***P<0.001, compared with the non-severe patients; ^ *P<*0.05, compared with the patients without liver diseases.

**Table 4:** Complications of 253 COVID-19 patients

Complications (No., %)	Total (N=253)	Non-severe (N=19)	Severe (N=58)	Without liver disease (N=202)	With liver disease (N=51)
One or more complications	69 (27.3)	35 (17.9)	34 (58.6) ***	48 (23.8)	21 (41.2) ^
Drug-induced liver injury	16 (6.3)	6 (3.1)	10 (17.2) ***	10 (5.0)	6 (11.8)
Hematological changes	34 (13.4)	15 (7.7)	19 (32.8) ***	21 (10.4)	13 (25.5) ^^
Anemia	13 (5.1)	5 (2.6)	8 (13.8) **	8 (4.0)	5 (9.8)
Hyperlipidaemia	11 (4.3)	8 (4.1)	3 (5.2)	7 (3.5)	4 (7.8)
Hypokalemia	9 (3.6)	4 (2.1)	5 (8.6) *	4 (2.0)	5 (9.8) ^^
Thrombus	3 (1.2)	0 (0)	3 (5.2) **	1 (0.5)	2 (3.9) ^
Cardiovascular injury	15 (5.9)	4 (2.1)	11 (19.0) ***	8 (4.0)	7 (13.7) ^^
Respiratory injury	14 (5.5)	2 (1.0)	12 (20.7) ***	8 (4.0)	6 (11.8) ^
Infection	12 (4.7)	5 (2.6)	7 (12.1) **	10 (5.0)	2 (3.9)
Lung injury	8 (3.2)	5 (2.6)	3 (5.2)	4 (2.0)	4 (7.8) ^
Genitourinary injury	10 (4.0)	3 (1.5)	7 (12.1) ***	6 (3.0)	4 (7.8)
Steroid-induced diabetes	2 (0.8)	0 (0)	2 (3.4) **	1 (0.5)	1 (2.0)
Anaphylaxis	6 (2.4)	3 (1.5)	3 (5.2)	4 (2.0)	2 (3.9)
Psychoneurotic symptoms	4 (1.6)	3 (1.5)	1 (1.7)	4 (2.0)	0 (0.0)
Electrolyte disturbance	3 (1.2)	0 (0)	3 (5.2) **	2 (1.0)	1 (2.0)

*P<0.05, **P<0.01, ***P<0.001, compared with the non-severe patients; ^ *P<*0.05, ^^ *P<*0.01, compared with the patients without liver diseases. Drug-induced liver injury was defined as that the patients were administrated one or more drugs which were proved might cause liver injuries, no abnormalities of liver function indicators of these patients were found before taking these drugs, however, abnormal increases of AST and ALT levels occurred during the drug treatment period, and the liver function improved after withdrawal of these drugs.

**Table 5:** Serological hepatic function index of 163 of the 253 COVID-19 patients within 14 days after discharge

Index	Average ± SD				Outlier ratio-N (%)			
	Control (N=152)	Patients (N=163)	Non-severe (N=117)	Severe (N=46)	Control (N=152)	Patients (N=163)	Non-severe (N=117)	Severe (N=46)
Female-N (%)	/	/	/	/	79 (52.0)	83 (50.9)	65 (55.6)	18 (39.1)
Age (years)	47.7±10.3	50.5±14.4*	47.9±14.7	57.4±11.0^^^	/	/	/	/
TP (g/L), Ref: 65-85	73.7±3.6	72.6±4.8*	73.3±4.7	70.9±4.8^^	3 (2.0)	8 (4.9)	4 (3.4)	4 (8.7)
ALB (g/L), Ref: 35-52	46.2±2.7	44.6±3.5***	45.2±3.2	43.2±3.7^^	2 (1.3)	3 (1.8)	2 (1.7)	1 (2.2)
GLB (g/L), Ref: 20-40	27.5±3.2	27.9±3.6	28.0±3.8	27.7±3.1	1 (0.7)	1 (0.6)	1 (0.8)	0 (0.0)
A/G, Ref: 1.5-2.5	1.7±0.3	1.6±0.2*	1.6±0.2	1.6±0.2	26 (17.1)	52 (31.9) **	31 (26.5)	21 (45.7)
ALT (U/L), Ref: women 7-40; men 9-50	22.9±19.7	36.3±34.4***	35.8±36.8	37.8±27.6	16 (10.5)	36 (22.1) **	24 (20.5)	12 (26.1)
AST (U/L), Ref: 15-40	22.3±12.2	24.1±15.4	24.2±17.3	23.7±9.3	38 (25.0)	32 (19.6)	26 (22.2)	6 (13.0)
AST/ALT, Ref: 0.5-1.5	1.18±0.44	0.84±0.37***	0.86±0.39	0.79±0.32	33 (21.7)	34 (20.9)	24 (20.5)	10 (21.7)
GGT (U/L), Ref: women 7-45; men 10-60	31.9±29.0	46.2±32.2***	41.3±27.7	60.0±38.7^^↑	20 (13.2)	52 (31.9) ***	28 (23.9)	24 (52.2) ^^^
ALP (U/L), Ref: 40-130	65.2±16.2	97.7±58.9***	100.3±59.2	91.3±58.3	4 (2.6)	71 (43.6) ***	57 (48.7)	14 (30.4) ^

*P<0.05, **P<0.01, ***P<0.001, compared with the control group; ^*P<*0.05, ^^*P<*0.05, ^^^*P<*0.05, compared with the non-severe group. The symbol ↑ respected the value was significantly higher or than the normal reference range.

**Table 6:** Liver function recovery of 46 of 253 COVID-19 patients during the 2 months' follow-up period after discharge

	Average ± SD				Outlier ratio (%)			
	1^st^	2^nd^	3^rd^	4^th^	1^st^	2^nd^	3^rd^	4^th^
Time (days)	18.5±15.9	27.9±13.8**	33.6±14.4***	39.8±15.2***	/	/	/	/
TP (g/L)	72.5±5.2	72.8±3.4	72.6±4.3	73.7±4.9	1(2.2)	0(0.0)	3(6.5)	1(2.2)
ALB(g/L)	45.4±3.3	46.1±3.0	46.3±3.1	46.8±3.2*	1(2.2)	1(2.2)	1(2.2)	3(6.5)
GLB (g/L)	27.1±4.5	26.7±3.8	26.3±4.4	26.8±4.2	1(2.2)	1(2.2)	3(6.5)	2(4.3)
A/G	1.7±0.3	1.8±0.3	1.8±0.4	1.8±0.3	8(17.4)	7(15.2)	10(21.7)	8(17.4)
ALT (U/L)	39.0±52.5	26.4±23.7	25.6±19.7*	26.4±19.8	12(26.1)	9(19.6)	10(21.7)	3(6.5) *
AST (U/L)	28.1±26.3	22.4±12.2	22.0±9.2	21.6±8.2*	13(28.3)	10(21.7)	8(17.4)	3(6.5) **
AST/ALT	1.0±0.5	1.1±0.5	1.1±0.5	1.0±0.5	8(17.4)	8(17.4)	21(45.7) **	13(28.3)
GGT (U/L)	37.3±32.5	30.5±28.1	25.9±17.4*	29.7±20.4	9(19.6)	5(10.9)	5(10.9)	4(8.7)
ALP (U/L)	74.0±47.8	48.9±32.9**	55.4±41.5*	47.7±24.1**	20(43.5)	24(52.2)	28(60.9)	21(45.7)

*P<0.05, **P<0.01, ***P<0.001, compared with the first detection.

## Abbreviations

SARS-Cov-2: severe acute respiratory syndrome coronavirus 2
COVID-19: coronavirus disease-2019
TP: total protein
ALB: albumin
GLB: globulin
ALT: alanine aminotransferase
AST: aspartate aminotransferase
GGT: gamma-glutamyl transferase
ALP: alkaline phosphatase
LDH: lactic dehydrogenase
CK: creatine kinase
CK-MB: creatine kinase isoenzyme
α-HBDH: alpha-hydroxybutyric dehydrogenase
Cr: creatinine
UA: uric acid
CO2CP: carbon dioxide combining power
β2-MG: and beta2-microglobulin
FT3: free triiodothyronine
FT4: free thyroxine
TSH: thyroid stimulating hormone
TPO: thyroid peroxidase
TG: thyroglobulin
Hs-CRP: high-sensitivity C-reactive protein
PCT: procalcitonin
IL-6: interleukin-6
IgM: immunoglobulin M
SAA: serum amyloid A
TC: total cholesterol
ApoAI: apolipoprotein AI
HDL-C: high density lipoprotein cholesterol
LP(a): lipoprotein a
HCY: homocysteine
Glu: glucose
WBC: white blood cell
NEUT: neutrophil
LYM: lymphocyte
MONO: monocyte
EOS: eosinophils
BASO: basophilic granulocyte
RBC: red blood cell
HCT: hematocrit
MCV: mean corpuscular volume
RDW-SD: red blood cell distribution width- standard deviation
RDW-CV: RDW-coefficient of variation
HGB: hemoglobin
MCH: mean corpuscular hemoglobin
MCHC: mean corpuscular hemoglobin concentration
PLT: platelet
MPV: mean platelet volume
PDW: platelet distribution width
P-LCR: platelet-large contrast ratio
