# Supplementary material for: Liver function recovery of COVID-19 patients after discharge, a follow-up study
Source: Int J Med Sci. 2021 Jan 1;18(1):176–86. doi: 10.7150/ijms.50691 (PMC7738966; doi:10.7150/ijms.50691)
Supplement: Supplementary file 1 — Supplementary figures. [file ijmsv18p0176s1.pdf]

## Supplementary data

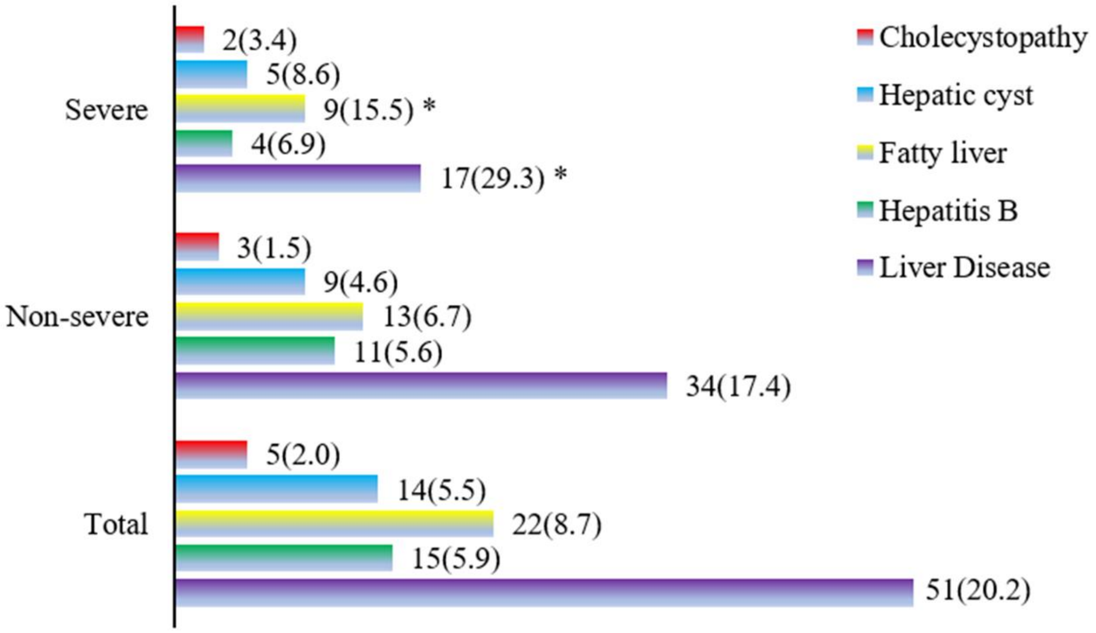


**Figure S1. Patients had liver diseases before they acquired COVID-19.** The Chi-square test (Monte Carlo Sig. T2) were used to compare the intergroup differences. *P<0.05, compared with non-severe group.

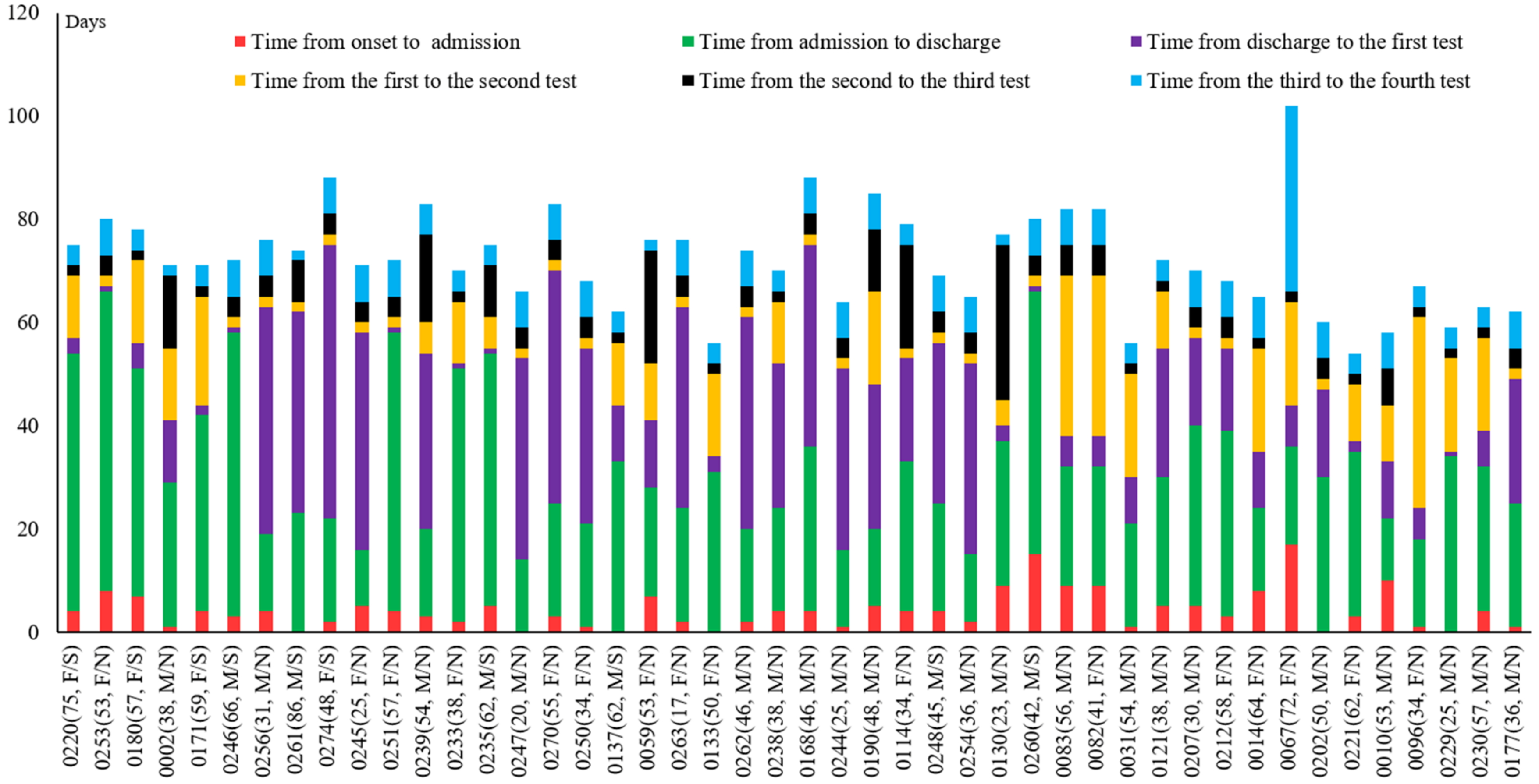


**Figure S2. Clinical characteristic and sampling information of the 46 patients.** The age of all 46 patients were 46.8±15.3 (range: 17-86; median: 48) years; of them, 20 (43.5%) were women, and 10 (21.7%) had severe condition during hospitalization. The x-ray was the hospitalization number (age, gender/severity), N=non-severe, S=severe, F=female, M=male.
